# A hierarchical Bayesian framework for inferring mitochondrial clonal selection from single-cell data

**DOI:** 10.21203/rs.3.rs-8490828/v1

**Published:** 2026-02-12

**Authors:** Aoqi Wang, Yanfei Wang, Xiaona Liu, Qing Wang, Sen Guo, Jianguo Wen, Xiaobo Zhou, Qianqian Song

**Affiliations:** 1 West China Biomedical Big Data Centre, West China Hospital, Sichuan University, Chengdu, Sichuan 610041, PR China; 2 Department of Health Outcomes and Biomedical Informatics, College of Medicine, University of Florida, FL, 32611, USA; 3 Center for Computational Systems Medicine, McWilliams School of Biomedical Informatics, The University of Texas Health Science Center at Houston, Houston, TX, 77030, USA; 4 Department of Cancer Biology, Wake Forest School of Medicine, Winston Salem, NC, 27101, USA.

**Keywords:** mitochondrial genetic heterogeneity, mitochondrial clonal selection, hierarchical bayesian modeling, selection pressure estimation, genotype–phenotype relationships, mitochondrial-driven pathogenesis

## Abstract

Mitochondrial genetic heterogeneity arises from the accumulation of somatic mitochondrial DNA (mtDNA) mutations within individual cells, generating intracellular clonal populations whose selective dynamics in disease remain poorly characterized. Here, we present MitoBayes, a hierarchical Bayesian framework that jointly models mitochondrial clonal lineage structure, allele frequency variation, and single-cell disease-relevant phenotypic burdens to infer clone-specific selection pressures. Extensive benchmarking demonstrates that MitoBayes accurately recovers ground-truth selection coefficients across a wide range of genetic heterogeneity, data sparsity, and lineage complexity scenarios. Application of MitoBayes to single-cell atlases of Alzheimer’s disease (AD) cortex, treatment-naïve non–small-cell lung cancer (NSCLC), and chemotherapy-resistant small-cell lung cancer (SCLC) revealed distinct, disease-specific patterns of mitochondrial clonal selection. These include selective expansion of high-risk mitochondrial clones associated with disruption of PVALB interneuron homeostasis in AD; disease-driven clonal remodeling in cycling T/NK cells from NSCLC tumors characterized by increased mitochondrial biogenesis and impaired immune regulatory programs; and preferential enrichment of a tumor-associated MT-ATP6 (m.8859A>G) clone linked to metabolic adaptation and platinum resistance in SCLC. Pan-cancer survival analyses further confirmed the clinical relevance of elevated MT-ATP6 activity, which was associated with inferior chemotherapy outcomes. Additionally, in hepatocellular carcinoma (HCC), a dominant m.2356C>G clone correlated with POLR2A activation and widespread transcriptional amplification, consistent with a mitochondria–nucleus signaling axis contributing to adverse prognosis in this cancer type. Collectively, these findings establish MitoBayes as a robust statistical framework linking mitochondrial genetic diversity to disease phenotypes and highlight mitochondrial clonal selection as a mechanistically and clinically actionable target for therapeutic and diagnostic development.

## INTRODUCTION

Mitochondria (MT) are the central hubs of cellular energy metabolism, redox homeostasis, and apoptosis regulation. Their functional equilibrium is essential for cellular survival and health, whereas dysfunction contributes directly to major diseases, including aging, neurodegenerative disorders, metabolic syndrome, and cancer^1, 2^. The compact, multicopy mitochondrial genome (mtDNA) accumulates somatic mutations stochastically with age and disease, producing heterogeneous cell populations composed of distinct mitochondrial clones^3^. These mutations are subject to microenvironmental selection, which shapes clonal dynamics by favoring advantageous variants and eliminating detrimental ones^4^. Moreover, mismatches between nuclear and mitochondrial genetic backgrounds can further drive somatic evolution of the mitochondrial genome^5^. This interplay between mutation and selection governs mitochondrial diversity^6^ and is thought to influence disease susceptibility, progression, and cell-to-cell heterogeneity across many pathologies^7^. Therefore, an in-depth understanding of how mtDNA variants affect disease is essential and requires the simultaneous consideration of mutational heterogeneity, cell-specific metabolic contexts, and the selection pressures acting upon them.

Despite growing recognition of the role of mitochondrial heterogeneity in disease^8–11^, several major limitations have historically hindered mechanistic undrestanding and clinical translation. A key barrier is the lack of single-cell resolution in studies of mtDNA heteroplasmy. Most analyses rely on bulk sequencing, which averages signals across thousands of cells and masks cell-to-cell variability. As a result, it is not possible to determine whether mutations are uniformly distributed across the population or concentrated within distinct clonal lineages that may exert unique functional effects. Morevoer, technical challenges in detecting mtDNA mutations at the single-cell level further complicate the field. While single-cell RNA-seq incidentally capture mitochondrial reads, coverage is typically sparse and uneven^12^. In addition, nuclear mitochondrial pseudogenes (NUMTs), RNA editing artifacts, and low read counts introduce errors and inflate noise in allele fraction estimates^13^. Although targeted enrichment approaches such as MAESTER^12^ and advanced filtering methods like mgatk^14^ can mitigate some of these issues, reliable identification of mitochondrial variants in individual cells remains difficult, particularly in heterogeneous tissue samples. Another limitation lies in reconstructing mitochondrial clonal lineages, which requires specialized tools distinct from those used for nuclear genomes. Unlike nuclear DNA, mitochondria are multicopy, lack recombination, and undergo stochastic bottlenecks. These features violate the assumptions of conventional lineage-tracing methods, which are not designed to handle polyploid, heteroplasmic genomes. Although recent methods such as MQuad^15^ and MERLIN^16^ have made progress, robust and accurate reconstruction of mitochondrial clones in complex tissues continues to pose a challenge. Finally, there has been no quantitative framework to directly link mitochondrial clones with cellular disease phenotypes. Existing workflows are largely sequential, in which variants are called, clones are inferred, and only afterward are associations tested with transcriptional states or other phenotypes. This design fails to capture the interdependence of genotype, lineage, and phenotype imposed by disease-specific remodeling, thereby limiting statistical power to identify rare but functionally relevant clones.

To address these limitations, we developed MitoBayes, a hierarchical Bayesian framework for joint modeling of mtDNA variant patterns, clonal heteroplasmy, and cellular disease states at single-cell resolution. MitoBayes integrates three complementary data layers: (i) per-cell mtDNA variant allele frequencies, (ii) inferred mitochondrial clonal lineages, and (iii) cell-specific disease scores derived by integrating GWAS-based polygenic signatures with single-cell transcriptomes. The model resolves intracellular heteroplasmy by linking per-cell allele frequencies with clone lineage through a zero-inflated Gaussian mixture component, thereby accounting for both the excess of zero-variant cells and the continuous distribution of heteroplasmy in others. By jointly modeling genotype, lineage, and phenotype, MitoBayes directly estimates a selection coefficient for each clone, providing a quantitative measure of how strongly that clone is favored or suppressed in diseased cells. Its hierarchical Bayesian structure, coupled with a hybrid MCMC inference scheme, ensures robustness to the sparsity and noise inherent in single-cell datasets. We applied MitoBayes to diverse disease contexts, including Alzheimer’s disease (AD) brains to investigate neuronal vulnerability, treatment-naïve non-small-cell lung cancer (NSCLC) tumors to assess immune cell dysfunction, chemotherapy-resistant small-cell lung cancer (SCLC) to explore clonal mitochondrial adaptations underlying chemoresistance, and hepatocellular carcinoma (HCC) to delineate mitochondria–nucleus interactions associated with poor prognosis. Across these settings, MitoBayes revealed that disease environments selectively remodel the mitochondrial genome, with specific clones expanding or contracting in concert with disease-associated cellular states. These findings not only advance our understanding of mitochondrial clonal selection in pathogenesis, but also highlight its potential as both a biomarker of disease progression and a potential target for therapeutic intervention.

## RESULTS

### MitoBayes framework quantifies clone-specific selection pressure

**Fig. 1:**
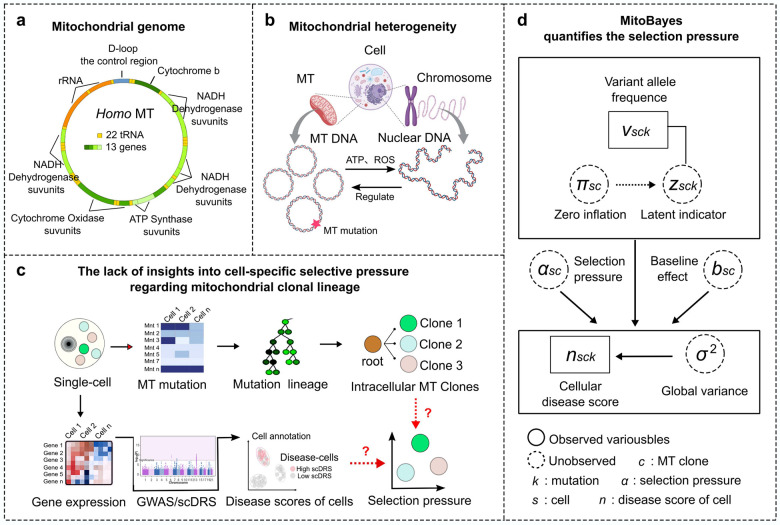
Schematic overview of the MitoBayes framework. **a**-**b** Mitochondrial heterogeneity arising from diverse mtDNA mutations within cells. **c** Analytical workflow integrating single-cell mitochondrial mutation detection, clonal lineage. **d** Hierarchical Bayesian model structure for estimating selection pressures acting on mitochondrial clones.

To systematically reveal how mitochondrial clonal heterogeneity influences cellular adaptation to disease, we developed an integrative framework to quantify the selection pressure acting on mitochondrial clones in association with cellular disease phenotypes. Fig. 1a illustrates the biological context: mitochondria have their own 16.6Kb circular DNA genome with a high mutation rate, and play a crucial role in energy production through oxidative phosphorylation, as well as regulating apoptosis. Each cell contains many mitochondrial genomes that accumulate diverse mutations over time, resulting in pronounced mtDNA heterogeneity both within and between cells (Fig. 1b). This heteroplasmy forms the basis of clonal diversity with subpopulations of mitochondria defined by certain mutations, which may have distinct functional consequences in health and disease. Fig. 1c provides an overview of the MitoBayes framework. From single-cell RNA-seq, we simultaneously obtained mtDNA mutations and nuclear gene expression in thousands of individual cells. We first identify mitochondrial clones by grouping cells that share unique mtDNA mutation signatures (and infer their clonal lineage relationships using the MERLIN^16^ algorithm). Each cell is annotated with a disease score, a numerical value representing the association of that cell’s transcriptome with a disease of interest, which is calculated via scDRS^17^ (single-cell Disease Relevance Score) that links single-cell gene expression to disease-risk gene sets from GWAS summary statistics. Fig. 1d summarizes the core modeling strategy of MitoBayes to quantify clone-specific selection pressure. MitoBayes employs a hierarchical Bayesian framework that integrates, for each cell, its mtDNA variant profile, inferred mitochondrial clonal lineage, and a quantitative disease-associated score. The model characterizes the distribution of disease scores as a function of mitochondrial clonal identity, while explicitly accounting for heteroplasmy and data sparsity, and yields posterior estimates of clone-specific selection coefficients. A positive selection coefficient indicates that a clone tends to reside in high disease-score cells (i.e. under positive selection in the disease environment), whereas a negative value indicates the clone is counter-selected (enriched in low-score or healthy cells). Through this framework, MitoBayes enables high-resolution prioritization of mitochondrial clones and mutations that are most functionally relevant to disease, offering new insights into the adaptive landscape of mitochondrial genetics in cellular pathogenesis.

### MitoBayes accurately and robustly recovers mitochondrial clone-specific selection pressure

**Fig. 2:**
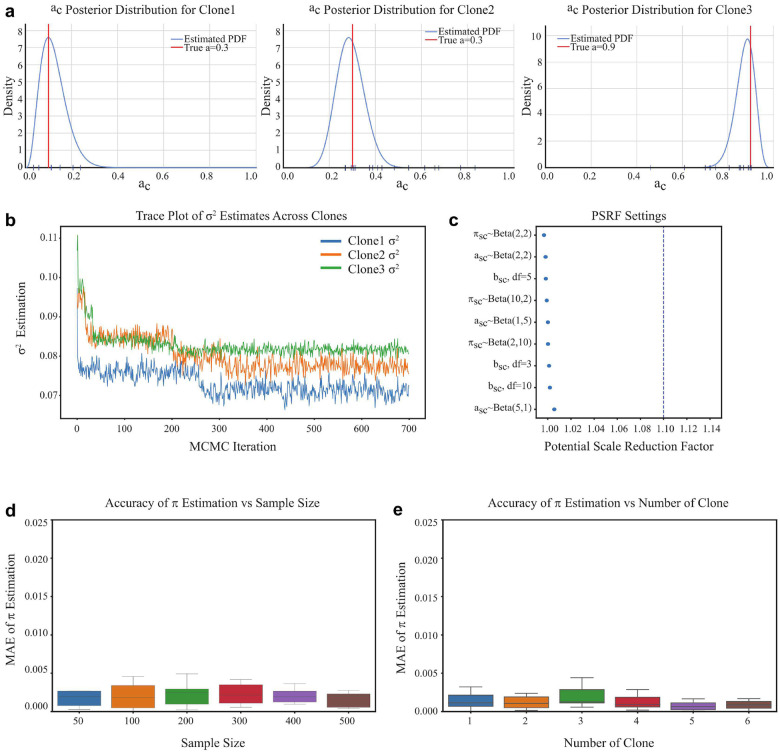
Model evaluation results of MitoBayes. **a** Posterior distributions of the selection parameters of three clones. The blue ones represent the estimated probability density functions, and the red ones represent the true values. **b** Trajectory diagram of the variance estimation of each clone residual. **c** The values of the latent scale Reduction factor (PSRF) under different prior Settings. **d** Estimation accuracy of the zero expansion probability with different sample sizes (number of cells). **e** Estimation of the zero expansion probability with different number of clones.

To evaluate the accuracy and robustness of our MitoBayes model in recovering clone-specific selection pressure, we conducted controlled simulations using three synthetic mitochondrial clones. Within the cell s, each mitochondrial clone c was assigned a distinct ground truth value for the selection parameter $a_{sc}$, representing varying degrees of selection pressure: low ($a_{sc}=0.1$), medium ($a_{sc}=0.3$), and high ($a_{sc}=0.9$). Fig. 2a presents the posterior density plots of the selection parameters $a_{sc}$ estimated from the MH-within-Gibbs sampling algorithm. The posterior distributions for each clone are centered sharply at their respective ground truth values, demonstrating that the model estimation accurately captures the underlying selection pressure. Even in the low-signal setting (Clone 1, $a_{sc}=0.1$), where the contribution of selection pressure to cellular disease is minimal, the posterior distribution remains well-calibrated and tightly concentrated, without significant spread or scattered. The sharpness of the density peaks and the alignment with true values across all clones also indicate that the joint modeling of variant allele frequency ($v_{sck}$) of each mutation (k) in each clone within each cell and cellular disease score of each cell provides sufficient information to disentangle selection pressure from background noise and stochasticity. To ensure convergence and stability of the posterior sampler, we examined the trace plots of the residual variance parameter $\sigma^{2}$ across Gibbs iterations for the same three representative clones. Fig. 2b shows that all chains converge rapidly from their initial values and reach a stable equilibrium without visible trends, oscillations, or divergence. The trace plots exhibit good mixing behavior and remain within tight bounds, demonstrating that the Gibbs update step for is highly efficient. The variance parameter plays a key role in modeling the Gaussian noise component of the cellular disease score distribution, and poor mixing could distort inference on other dependent parameters like $a_{sc}$ and $b_{sc}$. The stable trace patterns across clones suggest that the posterior distribution is not overly sensitive to initial conditions, sample size, or signal strength, further supporting the robustness of the model architecture.

We next assessed global convergence diagnostics for key model parameters ($\pi_{sc},a_{sc},b_{sc}$) by computing the Potential Scale Reduction Factor (PSRF), across multiple MCMC chains and prior settings. Here $\pi_{sc}$ is the clone-specific zero-inflation probability. Fig. 2c summarizes the PSRF values obtained under different configurations for the priors of $\pi_{sc}$, $a_{sc}$ and $b_{sc}$. Specifically, we varied the hyperparameters of Beta distributions for $\pi_{sc}$ and $a_{sc}$ including symmetric (Beta(2,2)), left-skewed (Beta(1,5)), and right-skewed (Beta(5,1)) configurations, as well as the degrees of freedom for the normal prior (e.g., N(0,0.5), N(0,2)). Across all tested conditions, the PSRF values remained below the threshold of 1.1, with most clustering closely around 1.0, indicating excellent mixing and convergence properties of the Gibbs algorithm. Notably, even under strongly skewed prior distributions or heavier-tailed settings, the PSRFs did not deteriorate, suggesting that the posterior inference remains robust to prior misspecification. This is particularly relevant for real-world applications where prior beliefs about parameter distributions may be uncertain. These results underscore the reliability and flexibility of our Bayesian framework in diverse biological and modeling scenarios.

To evaluate the influence of data availability on estimation precision, we assessed the mean absolute error (MAE) of the posterior estimate of zero-inflation probabilities ($\pi_{sc}$) across a range of sample sizes. In this setting, we generated datasets of varying sample sizes (number of cell: 50–500), each with 200 mutations per sample, and repeated the experiment ten times per size to measure variability. As illustrated in Fig. 2d, the MAE of estimation decreases monotonically as sample size increases, from roughly 0.0025 at 50 cells to below 0.0015 at 500 cells. The trend reflects the expected Bayesian learning effect, where more data contributes to sharper posteriors and reduced uncertainty. To assess how heterogeneity in mitochondrial clonal lineage affects the estimation of $\pi_{sc}$, we simulated datasets containing different numbers of mitochondrial clones, ranging from 1 to 6. Each clone has distinct values of $\pi_{sc}$ and $b_{sc}$. We then computed the MAE of posterior estimates for over repeated simulations. As shown in Fig. 2e, the MAE of estimation remained stable and consistently low across increasing numbers of clones, suggesting that our model effectively isolates clone-specific signals without being adversely affected by the added structural complexity. This finding highlights the scalability and robustness of our approach, particularly in settings involving mitochondrial subclonality and heterogeneous selection pressures.

### MitoBayes uncovers mitochondrial lineage shifts in Alzheimer’s disease

To demonstrate the utility of MitoBayes, we applied it to investigate mitochondrial clonal selection in Alzheimer’s disease (AD). We analyzed single-cell transcriptomic data from human prefrontal cortex, which includes thousands of nuclei from 12 AD patients and 9 age-matched healthy controls^18^. Using the MitoBayes framework, each cell was assigned an AD association score, derived from polygenic risk signals identified in AD GWAS data (GCST007511^19^, see Methods: Disease relevance score). In parallel, mtDNA variants were detected from the same dataset, enabling the identification of three dominant mitochondrial clones shared across individuals. These mtDNA variants, together with cell-level AD association scores, were integrated into the MitoBayes model to estimate clone-specific selection pressures, thereby quantifying the contribution of mitochondrial clonal dynamics to AD pathogenesis.

**Fig. 3:**
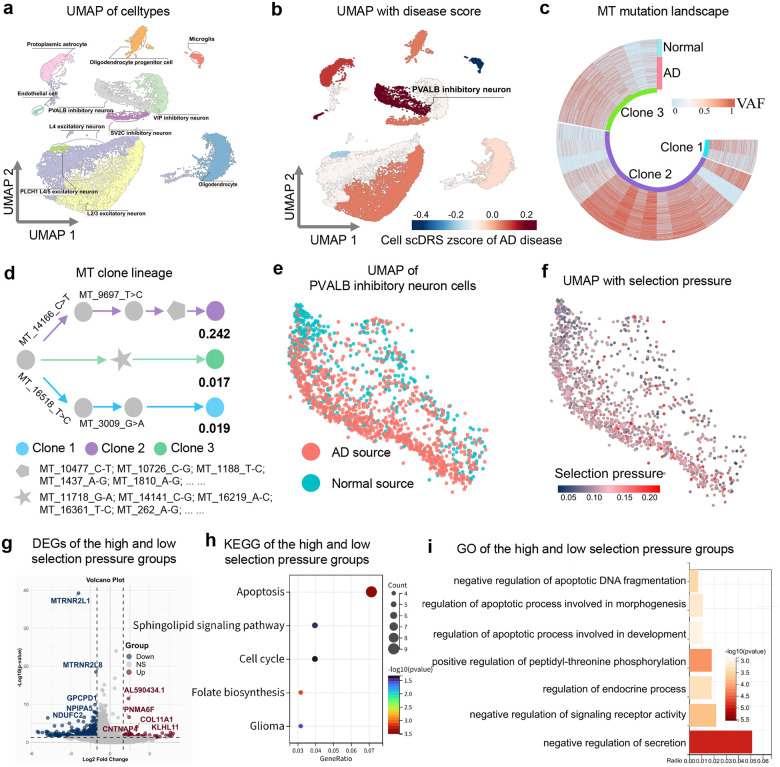
Mitochondrial clonal lineage remodels neuronal adaptation in AD. **a** UMAP plot showing cell type distribution in GSE157827 single-cell data. **b** Distribution of disease scores across cell types. **c** mtDNA mutation landscape in PVALB inhibitory neurons. **d** Mitochondrial clonal lineage tree in PVALB neurons. **e** Distribution of AD and normal PVALB neurons. **f** Mapping of mitochondrial selection pressure at single-cell resolution. **g** Differential expression analysis for high versus low selection pressure cells. **h-i** KEGG and GO enrichment of pathways associated with high selection pressure.

Unsupervised cell clustering and UMAP visualization (Fig. 3a) revealed the distribution of neuronal and glial subtypes. Mapping the AD association score across cells (Fig. 3b) showed that, among neuronal populations, PVALB-positive inhibitory interneurons exhibited the highest AD association. This indicates PVALB inhibitory neurons as a particularly vulnerable subtype in AD, consistent with prior studies of interneuron dysfunction as a critical driver of neuronal network dysfunction and cognitive decline in AD mouse models^20^. Focusing on these PVALB inhibitory neurons, we constructed the mitochondrial mutational landscape (Fig. 3c) and clonal lineage tree (Fig. 3d). Three predominant mitochondrial clones (denoted Clone 1–3) were identified across both AD and control individuals. Although all clones were present in both groups, their relative abundances and heteroplasmy patterns differed markedly, suggesting potential disease-specific selection. Subclustering of PVALB inhibitory neurons by disease status (Fig. 3e) showed that AD-derived PVALB inhibitory neurons were largely segregated from healthy control neurons in transcriptomic space, indicating substantial AD-related transcriptional remodeling within this cell type. We next mapped the quantified mitochondrial selection pressures in PVALB inhibitory neurons (Fig. 3f), which revealed that PVALB inhibitory neurons from AD patients exhibited systematically higher mitochondrial selection pressure values than healthy controls. Notably, Clone 2 emerged as the dominant high-pressure clone, with an estimated selection coefficient of 0.24). These findings suggest that certain mitochondrial variants and clones undergo preferential expansion in AD-vulnerable neurons, potentially exacerbating to the cells’ dysfunction.

To assess the functional consequences of clonal selection, we performed differential gene expression analysis between PVALB inhibitory neurons under high versus low mitochondrial selection pressure. Fig. 3g highlights the top differentially expressed genes. Notably, cells under high mitochondrial selection pressure showed upregulation of *AL590434.1*, an uncharacterized transcript implicated in sphingolipid signaling, a pathway central to neuronal membrane structure and signaling^21^. *PNMA6F* (Paraneoplastic Ma antigen family member 6F) was also upregulated; while its specific function remains undefined, other PNMA family proteins are involved in synaptic integrity and neuronal membrane stability^22^, suggesting potential perturbations in neuronal connectivity. In contrast, two genes, *MTRNR2L1* and *MTRNR2L8*, nuclear paralogs of the mitochondrial 16S rRNA gene that encode humanin-like peptides known to inhibit apoptosis and mitigate stress responses^23, 24^, were significantly downregulated in high-pressure cells. Their reduced expression suggests a loss of neuroprotective signaling, potentially increasing neuronal vulnerability to stress-induced cell death.

Pathway-level analyses corroborated these gene-level observations. KEGG pathway enrichment (Fig. 3h) revealed activation of apoptotic pathways, cell cycle checkpoints, and DNA damage responses in high-pressure PVALB inhibitory neurons cells. GO biological process enrichment (Fig. 3i) highlighted mitochondrial dysfunction-related terms, including impaired oxidative phosphorylation, altered mitophagy/autophagy, and broader cellular stress responses. Pathways regulating mitochondrial DNA replication and quality control were also perturbed, indicating that high selection pressure for certain mtDNA variants may coincide with compromised mitochondrial maintenance. Collectively, these results establish a quantitative link between mitochondrial clonal selection and neuronal transcriptomic remodeling in AD. Selective expansion of specific mtDNA clones in PVALB inhibitory neurons correlates with reduced neuroprotective capacity (via downregulation of humanin-like peptides) and increased activation of stress, injury, and cell death pathways. This mitochondrial-genetic influence may contribute to the intrinsic vulnerability of these neurons in AD, potentially exacerbating network hyperexcitability or loss of inhibitory tone in cortical circuits. By prioritizing specific mitochondrial clones and their downstream functional effects, the MitoBayes framework highlights candidate cell type- and organelle-level therapeutic targets for neurodegeneration.

### MitoBayes uncovers mitochondrial clonal selection underlying immune cell dysregulation

**Fig. 4:**
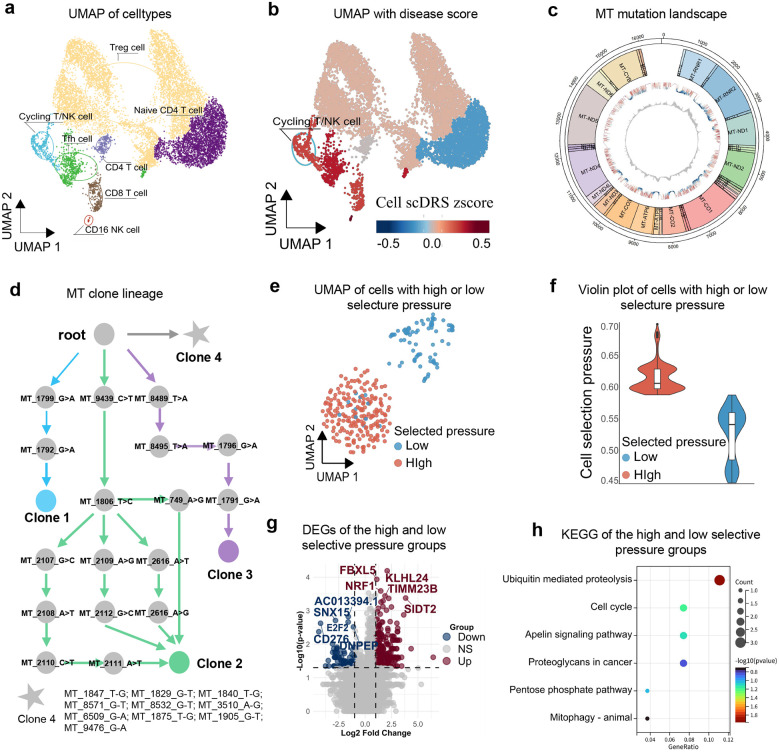
Disease-driven mitochondrial clonal selection disrupts immune cell homeostasis in lung cancer. **a** UMAP of immune cell types in NSCLC scRNA-seq data. **b** UMAP with NSCLC disease score per cell. **c** Landscape of mtDNA mutations in Cycling T/NK cells. **d** Reconstructed mitochondrial clone lineage tree. **e** UMAP of Cycling T/NK cells colored by selection pressure (high vs low). **f** Violin plot of selection pressure across cells. **g** Volcano plot showing differentially expressed genes between high and low selection pressure groups. **h** KEGG pathway enrichment of differentially expressed genes.

We next investigated mitochondrial clonal dynamics in the context of non-small-cell lung cancer (NSCLC), with a particular focus on the tumor-infiltrating immune cells. The NSCLC tumor microenvironment exerts profound metabolic and immunosuppressive pressures, often leading to T cell dysfunction or “exhaustion” that facilitates immune evasion^25^. We examined that whether selective expansion of mitochondrial clones within immune cells may contribute to, or reflect this dysfunctional state. Our analysis centered on a distinct subset of T cells known as Cycling T/NK cells, identified in single-cell RNA-seq data from NSCLC tumors by their proliferative signatures and transitional phenotype between T cells and natural killer (NK) cells^26^. These Cycling T/NK cells possess dual functional potential, combining the cytotoxic effector activity of CD8+ T cells with the proliferative expansion typical of NK cells^27, 28^. Within the NSCLC dataset, Cycling T/NK cells emerged as the most lung disease-associated immune subset. Specifically, Fig. 4a shows the UMAP distribution of immune cells from NSCLC tumors, and Fig. 4b maps NSCLC disease scores using lung cancer GWAS summary statistics. Among various T cell and myeloid subsets, Cycling T/NK cells exhibited the highest disease scores (see Methods: Disease relevance score), indicating they are strongly affected in the tumor microenvironment.

To ensure robustness of mitochondrial variant detection, we first confirmed adequate sequencing depth across the mitochondrial genome in this subset. Fig. 4c demonstrates uniform mtDNA coverage, validating reliable variant calling. Reconstruction of mitochondrial clonal lineage in Cycling T/NK cells, based on shared mtDNA mutations and lineage inference via MERLIN^16^, revealed four distinct mitochondrial clones (Fig. 4d). These clones coexisted within the tumor-infiltrating T/NK compartment, intriguing to assess whether specific clones were preferentially selected under tumor-induced stress. Applying MitoBayes, we estimated clone-specific selection coefficients and per-cell selection pressures. The analysis revealed a clear bifurcation among Cycling T/NK cells: a subset exhibited markedly elevated selection pressures associated with one mitochondrial clone, while others remained under neutral or no selection. Fig. 4e highlights the spatial distribution of high- versus low-selection-pressure cells on the UMAP, and Fig. 4f shows violin plots of their selection coefficient distributions. Importantly, high-selection-pressure cells clustered together in transcriptomic space, suggesting shared transcriptional remodeling. These findings indicate that mitochondrial clonal lineage exerts a measurable impact on immune cell state.

We next examined transcriptomic correlates of mitochondrial selection pressure. Differential gene expression analysis (Fig. 4g) revealed that high-pressure Cycling T/NK cells upregulated multiple stress- and quality-control related genes, including *KLHL24* (an E3 ubiquitin ligase involved in protein quality control), *SIDT2* (a lysosomal membrane protein mediating autophagy and nucleic acid transport), *FBXL5* (an iron-responsive E3 ligase linked to oxidative stress), and *NRF1*, a transcription factor that regulates mitochondrial biogenesis and respiratory function. The induction of *NRF1* is particularly notable, as it may represent a compensatory attempt to restore mitochondrial homeostasis under clonal stress^29^. Pathway enrichment analyses (Fig. 4h) revealed activation of ubiquitin-mediated proteolysis, mitophagy/autophagy, cell cycle regulation, and apoptotic pathways, consistent with a broad cellular stress response. Together, these results suggest that high-pressure Cycling T/NK cells engage survival-oriented transcriptional programs at the expense of effector functionality.

Collectively, our findings indicate that in NSCLC, expansion of a tumor-associated mitochondrial clone in Cycling T/NK cells fundamentally alters their biological state. Rather than maintaining a balance between proliferative expansion and cytotoxic effector activity, cells under strong mitochondrial selection pressure shift toward stress adaptation and survival, compromising their anti-tumor function. This observation extends emerging evidence that mitochondrial dysfunction drives T cell exhaustion in the tumor microenvironment^30, 31^, by identifying a specific mitochondrial genotype that confers survival advantage but simultaneously diminishes immune competence. Practically, these results suggest that mtDNA mutations can endow immune cells with stress resilience under tumor conditions, but at the cost of effective anti-tumor immunity. Such mitochondrial clonal remodeling thus represents a novel mechanism of immune evasion in cancer. Targeting this process, for example, by selectively eliminating or reprogramming high-pressure mitochondrial clones, may offer a therapeutic avenue to restore immune surveillance in NSCLC.

### Mitochondrial clonal remodeling underlies metabolic adaptation and chemotherapy resistance

**Fig. 5:**
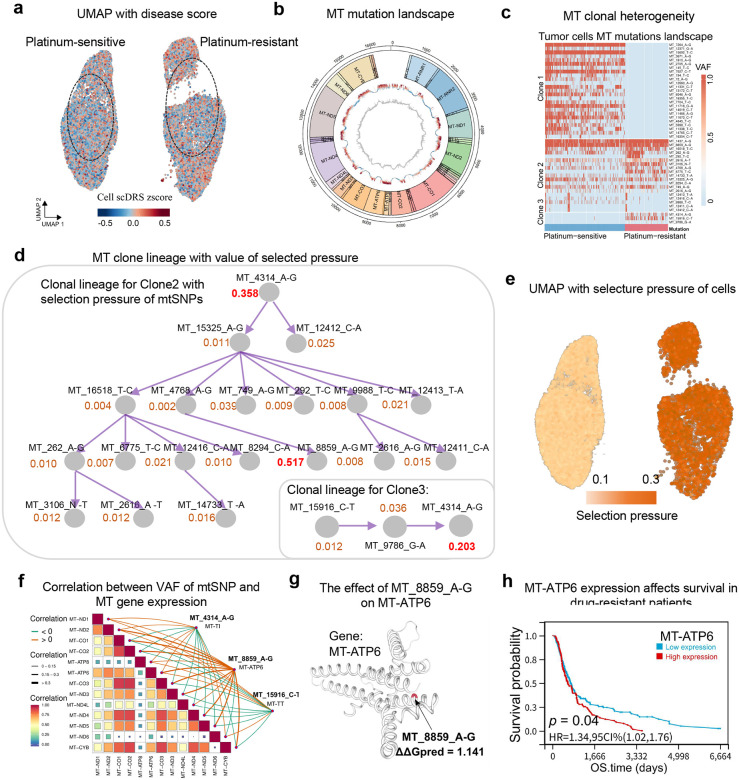
Mitochondrial clonal remodeling underlies metabolic adaptation and chemotherapy resistance in small-cell lung cancer. MitoBayes analysis of therapy-resistant SCLC. **a** UMAP projection of single-cell transcriptomes separates platinum-sensitive and platinum-resistant SCLC tumor cells. **b** Circular plot of mitochondrial mutation distribution across the mtDNA genome. **c** Heatmap displaying mitochondrial clonal heterogeneity between sensitive and resistant tumor cells. **d** Mitochondrial clonal lineage reconstruction with selection pressure values for key mtSNVs. **e** UMAP plot showing cell-wise mitochondrial selection pressure; resistant cells exhibit higher values. **f** Correlation matrix between variant allele frequency of major mtSNVs and mitochondrial gene expression. **g** Predicted structure of MT-ATP6, highlighting the MT_8859_A>G mutation and its effect on protein stability. **h** Kaplan–Meier survival analysis demonstrating that high MT-ATP6 expression predicts poorer prognosis in drug-resistant cancer patients.

We next investigated whether mitochondrial clonal selection contributes to chemotherapy resistance in small cell lung cancer (SCLC). SCLC is an aggressive malignancy in which initial platinum-based chemotherapy often elicits strong responses, but relapse with drug-resistance^32^. Herein, we analyzed a single-cell RNA-seq dataset of SCLC patient-derived xenografts, which included tumor cells from both platinum-sensitive and platinum-resistant samples^33^. This enabled a direct comparison of mitochondrial genomics between therapy-naïve and therapy-resistant cancer cell populations. UMAP visualizations of all tumor cells (Fig. 5a) revealed two distinct clusters corresponding to platinum-sensitive versus resistant cells, indicating substantial transcriptomic reprogramming during the development of chemoresistance. We next profiled the mitochondrial mutation landscape across these cells. Fig. 5b presents mtDNA variant positions and allele frequencies in sensitive vs resistant groups, revealing widespread mtDNA single-nucleotide variants (mtSNVs) across the mitochondrial genome. Fig. 5c further summarizes clonal heterogeneity with a heatmap of the major mitochondrial clones and their prevalence in sensitive vs resistant tumor cells. Notably, the resistant cells exhibited a distinct clonal lineage, in which certain clones that were rare in sensitive cells became dominant in the resistant population, suggesting these clones were selected during or after chemotherapy.

Using MitoBayes, we quantified clone-specific selection pressure in SCLC. Fig. 5d shows a reconstructed mitochondrial clone lineage tree with estimated selection pressure for key clones. Several mtDNA variants exhibited strong positive selection in resistant tumors, most notably a mutation at mtDNA position 8859 (MT_8859_A>G, an mtDNA base substitution from A to G at position 8859), which occurs in the *MT-ATP6* gene encoding a subunit of the ATP synthase complex. This MT_8859_A>G variant had the highest selection pressure (with an estimated selection coefficient of 0.517) among all variants, and was enriched specifically within the resistant cluster. Moreover, the heterogeneous mutation MT_4314_A>G (homogeneous Allele frequency of 1.24e-4 is from gnomAD; non-coding mutation) has a relatively high selection pressure (with an estimated selection coefficient of 0.358, and simultaneously as a genetic marker for clones 2 and 3) and is enriched as a somatic mutation in platinum-resistant SCLC. Consistently, mapping cell-wise mitochondrial selection pressures on the UMAP (Fig. 5e) revealed markedly elevated pressures in resistant versus sensitive cells, indicating strong disease-driven selection favoring this genotype during therapy resistance. To investigate why MT_8859_A>G confers a selective advantage, we correlated variant allele frequencies with nuclear-encoded mitochondrial gene expression. Fig. 5f shows that the mutation was strongly associated with upregulation of oxidative phosphorylation (*OXPHOS*) genes, suggesting an enhanced mitochondrial transcriptional program. Structural modeling of the ATP synthase complex incorporating the amino acid change induced by MT_8859_A>G predicted a modest increase in protein stability (ΔΔG ≈ +1.14 kcal/mol; Fig. 5g). While subtle, this gain could improve ATP synthase efficiency or stability. In the drug-stressed tumor microenvironment, even minor improvements in bioenergetic output may confer survival advantage, allowing resistant clones to outcompete therapy-sensitive populations^34, 35^. Supporting this, resistant SCLC cells exhibited higher mitochondrial membrane potential and ATP levels in independent assays^36^, consistent with a more oxidative, energy-producing phenotype.

To extend these findings, we analyzed clinical data from The Cancer Genome Atlas (TCGA) pan-cancer cohort to test whether mitochondrial ATP synthase activity correlates with patient outcomes. Kaplan–Meier analysis (Fig. 5h) showed that across multiple cancer types, patients with high *MT-ATP6* expression were associated with significantly poorer survival following therapy compared to tumors with lower *MT-ATP6* expression (log-rank *p* = 0.04, Hazard Ratio ≈ 1.35). This is consistent with previous studies that high expression of mitochondrial ATP synthase components correlates with aggressive, therapy-resistant disease^37, 38^. Together, these results demonstrate that mitochondrial clonal remodeling underlies metabolic adaptation and chemoresistance in SCLC. In particular, the expansion of MT_8859_A>G clone enhances mitochondrial bioenergetics, enabling tumor cells to withstand chemotherapy-induced stress and promote relapse. These findings identify a specific mitochondrial genetic mechanism of resistance and suggest that targeting adaptive mitochondrial pathways may offer a novel therapeutic strategy to prevent or overcome chemoresistance in SCLC.

### MitoBayes uncovers mitochondria-nucleus interaction underlying poor prognosis in HCC

**Fig. 6:**
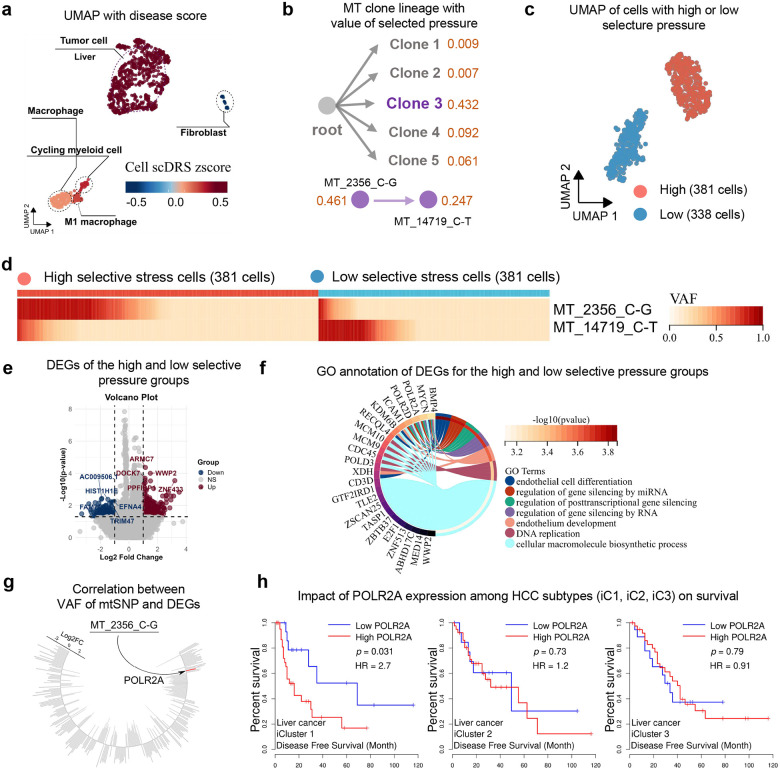
Mitochondrial clonal selection in HCC and its transcriptional and clinical relevance. **a** UMAP showing tumor cells with higher scDRS disease scores compared to non-tumor cells. **b** MitoBayes clonal lineage inference identifies five clones; Clone 3 exhibits the strongest selection (s = 0.432), driven by MT-2356_C>G (s = 0.461). **c** UMAP of tumor cells grouped by mitochondrial selection pressure, revealing transcriptomic divergence between high- and low-selection groups. **d** Heatmap of mtDNA variant frequencies shows MT-2356_C>G enriched in high-selection Clone 3 cells. **e** Volcano plot of differentially expressed genes (DEGs); upregulated genes include POLR2A, MCMs, CDK1, and EZH2; downregulated genes include ARID1A. **f** GO enrichment of DEGs highlights chromatin regulation, DNA replication, and transcriptional silencing. **g** MT-2356_C>G variant allele fraction positively correlates with POLR2A expression (p < 0.05). **h** Kaplan–Meier survival analysis in TCGA HCC shows that high POLR2A expression predicts poor outcome specifically in iCluster1 (HR = 2.7, p = 0.031).

We applied MitoBayes to single-cell data from Hepatocellular Carcinoma (HCC) tumors to delineate mitochondrial clones and quantify their selection pressures. UMAP visualization of cells colored by disease scores confirmed that tumor cells exhibited higher disease burden than non-tumor cells (Fig. 6a). Lineage analysis identified five distinct mitochondrial clonal lineages in the tumor, notably, one lineage (designated Clone 3) exhibited an exceptionally high selection pressure (with an estimated selection coefficient of 0.432), far surpassing the others (Fig. 6b), indicating strong positive selection. Expansion of Clone 3 appeared to be driven by a specific mtDNA variant, MT_2356_C>G, which itself showed a high selection coefficient (with an estimated selection coefficient of 0.461), marking it as the principal driver mutation (Fig. 6b). Stratification of tumor cells into high- versus low-selection pressure groups based on clonal lineage revealed clear separation in transcriptomic space (Fig. 6c). Consistently, the MT_2356_C>G mutation was almost exclusively found in the high-selection group, with Clone 3 cells harboring this variant at high variant allele fractions, whereas it was virtually absent in low-selection clone cells (Fig. 6d), supporting MT_2356_C>G as the driver of clonal dominance.

Differential expression analysis between high- versus low-selection tumor cells revealed widespread transcriptional reprogramming (Fig. 6e). Genes upregulated in high-selection tumor cells included those involved in transcriptional regulation, chromatin modification, and cell-cycle progression, while downregulated genes were enriched for chromatin remodeling and tumor-suppressive functions. For example, high-selection cells showed elevated expression of DNA replication licensing factors (MCM family, *CDK1*) and the epigenetic silencer *EZH2*, whereas the chromatin remodeling tumor suppressor *ARID1A* was among the most strongly downregulated. Gene Ontology enrichment analysis further highlighted chromatin organization, DNA replication, and transcriptional silencing as the top biological processes (Fig. 6f). These results indicate that the positively selected mitochondrial clone is associated with an epigenetically reprogrammed, highly proliferative transcriptional phenotype. To identify nuclear correlates of the MT_2356_C>G variant, we performed correlation analysis of variant allele frequency against gene expression. *POLR2A*, encoding the largest subunit of RNA polymerase II, emerged as the only nuclear gene significantly positively correlated with the MT_2356_C>G mutation burden (Fig. 6g). In cells with the highest variant loads, *POLR2A* expression was markedly elevated (p < 0.05), while no other genes showed significant associations. This reveals a potential mitochondria–nucleus interaction linking Clone 3 with activation of the transcriptional machinery. The clinical relevance of this axis was supported by TCGA integrative analysis of HCC. Prior stratification defined molecular subgroups (iClusters^39^) with distinct prognoses. The worst-prognosis subtype (iCluster 1) was characterized by immune exclusion, high proliferation, vascular invasion, and transcriptional amplification. Elevated *POLR2A* expression is consistent with this aggressive profile. Indeed, Kaplan–Meier analysis (Fig. 6h) demonstrated that within iCluster 1, high POLR2A expression predicted significantly worse disease-free survival (hazard ratio = 2.7, p = 0.031), whereas no prognostic effect was observed in other iClusters.

Together, these results suggest that mitochondrial clonal selection in HCC can reshape nuclear transcriptional programs central to tumor aggressiveness. The strong correlation between MT_2356_C>G and POLR2A expression supports a functional mitochondria-nucleus axis, whereby clonally expanded mtDNA mutations influence nuclear gene regulation through retrograde signaling or metabolic reprogramming. Given that iCluster 1 tumors are transcriptionally amplified and clinically aggressive, convergence of a positively selected mtDNA mutation with POLR2A-driven proliferation highlights direct crosstalk between mitochondrial genotype and nuclear oncogenic circuits. Thus, MT_2356_C>G may serve as both a marker and potential mediator of transcriptional regulation in liver cancer, with implications for biomarker development and therapeutic targeting.

## DISCUSSION

We developed MitoBayes, a quantitative framework that links mitochondrial clonal selection with cellular disease phenotypes at single-cell resolution. By integrating mitochondrial DNA variants, clonal lineage structures, and disease metrics derived from single-cell RNA-seq data, MitoBayes captures how pathological environments impose selection pressures on mitochondrial genomes. Extensive evaluations demonstrate that MitoBayes identifies selection pressures accurately and robustly across diverse settings. Applied to disease contexts, including AD, NSCLC, SCLC, and HCC, MitoBayes consistently identified mitochondrial clones under significant selection pressure, each with distinct biological consequences. These disease-specific case studies establish mitochondrial clones as both biomarkers of disease states and potential therapeutic targets. Moreover, the ability of MitoBayes to prioritize clones and mutations with functional and clinical relevance underscores its translational utility in dissecting mitochondrial contributions to disease pathogenesis.

Our results collectively demonstrate that mitochondrial clonal selection exerts disease-specific effects that extend beyond neutral drift, with broad implications for neuronal vulnerability, immune evasion, therapeutic resistance, and cancer progression. In AD, the preferential expansion of Clone 2 within parvalbumin-positive interneurons illustrates how mitochondrial variants actively shape neuronal susceptibility. Rather than being a by-product of aging, the systematic increase in selection pressure in AD interneurons was accompanied by transcriptional remodeling, including upregulation of stress and apoptotic pathways and downregulation of humanin-like peptides. These findings align with reports that interneuron dysfunction is an early driver of AD-related network failure^40^ and suggest that mitochondrial clonality erodes intrinsic neuroprotection, contributing to cortical disinhibition and cognitive decline^41^. In NSCLC, mitochondrial clonal selection was detected prominent in proliferative T/NK cells, where expansion of a high-pressure lineage coincided with induction of autophagy and proteostasis pathways and loss of cytotoxic signatures. This extends the concept of T cell exhaustion^42, 43^ by showing that specific mitochondrial genotypes can reinforce maladaptive transcriptional states, locking immune cells into stress-adapted but functionally impaired programs. Clinically, this implies that immune dysfunction in tumors may be sustained by selective mitochondrial lineages and may only be reversible if interventions directly target these maladaptive clones. In chemotherapy-resistant SCLC, MitoBayes revealed strong positive selection for a clone carrying the MT-ATP6 m.8859A>G mutation, previously reported in multiple diseases^44–48^. Structural modeling indicated that this variant enhances MT-ATP6 protein stability and oxidative phosphorylation, conferring tumor cells with a bioenergetic advantage that supports survival under chemotherapeutic stress. Consistent with prior reports linking ATP synthase activity to tumor aggressiveness, our findings establish that mitochondrial clonal expansion is an active driver of therapy resistance rather than a passive by-product. Clinically, high MT-ATP6 expression correlated with poor survival across cancer cohorts, positioning this clone as both a biomarker of poor prognosis and a metabolic vulnerability^49^. In HCC, MitoBayes identified a dominant clone carrying the MT_2356_C>G mutation, which showed strong positive selection and was tightly coupled to POLR2A upregulation and a proliferative, chromatin-rewired state. This demonstrates that mitochondrial clones can directly influence nuclear programs through retrograde signaling, linking mtDNA clonal dominance to dysregulated RNA polymerase II activity and transcriptional output. Importantly, tumors harboring this mitonuclear imbalance fell into the poorest-prognosis HCC, underscoring that mitochondrial clonal selection can reshape nuclear circuits and drive adverse clinical outcomes.

From a computational modeling perspective, MitoBayes addresses several key challenges in the field. First, it provides a quantitative framework for joint inference of mitochondrial genotype–phenotype relationships, overcoming limitations of prior descriptive approaches. Second, it explicitly resolves intracellular heteroplasmy by leveraging Bayesian hierarchical modeling, enabling robust estimation of selection pressures even from sparse single-cell data. Third, by integrating GWAS-derived disease relevance scores, MitoBayes establishes a bridge between single-cell transcriptomes and population-scale genetic risk, thereby situating mitochondrial clonal selection within broader disease contexts. Despite these advances, certain limitations should be acknowledged. The reliance on GWAS-derived association metrics may incompletely capture disease biology, particularly for conditions where genetic risk is polygenic or poorly characterized. Moreover, the observational nature of current datasets restricts causal inference, limiting the ability to distinguish between driver and passenger mitochondrial variants. Future extensions of MitoBayes could incorporate additional phenotypic modalities, such as metabolomics and spatial omics, to provide a more comprehensive view of mitochondrial function. Moreover, coupling MitoBayes with experimental perturbations, such as CRISPR-based mitochondrial genome editing, will provide solid validation of computational discoveries.

Together, our findings lay the groundwork for a mitochondrial precision medicine paradigm, in which mtDNA variants are considered not merely as genetic byproducts but as therapeutically actionable determinants of cellular state and disease trajectory. By applying MitoBayes across diverse diseases and tissue contexts, future studies can further elucidate how mitochondrial clonal dynamics shape health and pathology. Ultimately, this work opens new opportunities for targeted mitochondrial interventions, ranging from strategies to selectively eliminate pathogenic clones to approaches that restore healthy mitochondria–nucleus communication, thereby bridging mitochondrial biology with clinical applications in diagnostics and therapeutics.

## METHODS

### Data sources and processing

We analyzed three single-cell datasets, each representing a distinct biological system and question. For Alzheimer’s disease, we used single-nucleus RNA-seq data from human prefrontal cortex (GEO: GSE157827), which includes 21 samples (12 AD patients and 9 cognitively normal controls) profiled with 10x Genomics technology. For non-small-cell lung cancer (NSCLC), we used a published single-cell RNA-seq dataset of tumor-infiltrating immune cells (GSE164146), composed of 4 samples from 4 human non-small-cell lung cancer patients. For small-cell lung cancer (SCLC), we used single-cell transcriptomes from patient-derived xenograft tumors, including both platinum-sensitive and platinum-resistant samples (GSE138474). For hepatocellular carcinoma (HCC), we also analyzed data from GSE112271, focusing on primary liver tumor cells obtained from the same patient-derived xenograft model. Raw sequencing data were processed with standard pipelines: reads were aligned to the human reference genome (GRCh38) using STAR or Cell Ranger for transcript counts, and we extracted reads aligning to the mitochondrial genome (MT). Quality control was applied to retain high-quality cells; cells with low UMI counts, high mitochondrial transcript percentage (for nuclear data quality filtering), or insufficient coverage of mtDNA (for variant calling) were removed.

### Mitochondrial variant calling and clonal lineage inference

We identified mitochondrial DNA single-nucleotide variants (mtSNVs) for each cell by analyzing the aligned mtDNA reads. Specifically, we use MERCI method to detect mitochondrial mutations in single-cell transcriptome, and perform strict filtering to avoid false positives^50^. Mutations present in at least 50 cells will be retained. For each mutation detection result of each cell, it is required that the sequencing depth be greater than 10. We then inferred mitochondrial clones using the MERLIN^16^ algorithm for more rigorous lineage reconstruction. MERLIN formalizes the problem of inferring a concordant mitochondrial phylogeny nested within the cell lineage tree, accounting for heteroplasmy, and it uses a combinatorial optimization approach to find the best-fitting clone tree^16^. The clone lineage of each cell (which clone’s mutations it carries) was then used as an input for MitoBayes.

### Disease relevance score

We quantified a “disease association score” for each cell using the single-cell Disease Relevance Score (scDRS) method^17^. Briefly, scDRS leverages genome-wide association study (GWAS) results to score individual cells based on the expression of genes associated with a given disease or trait. For AD, we used a published AD GWAS data (Study: GCST007511, getting from GWAS Catalog database) to score neuronal cells^19^; scDRS essentially computes a polygenic risk score at the single-cell level by summing expression of risk genes weighted by their GWAS effect sizes, with statistical adjustments for gene set size and expression variability. For NSCLC and SCLC, we used GWAS hits for lung cancer to derive an “lung cancer score” for each cell (Study: GCST90479533, getting from GWAS Catalog database)^51^. For HCC, the GWAS data was getting from GCST90043858^52^. All scores were z-normalized across cells in a datase. These scDRS scores served as our quantitative phenotype of interest in the MitoBayes model (higher score means more disease-like cell; lower means more normal-like).

### MitoBayes model

We developed a Bayesian hierarchical model, termed MitoBayes, to estimate clone-specific selection pressure that quantify the association between mitochondrial clonal identity and cellular disease state at single-cell resolution. The model accounts for the heterogeneity in mutational effects as well as stochastic dropout inherent in single-cell mutation data through a zero-inflated Gaussian mixture formulation.

Let $n_{sck}$ denotes the disease relevance score for mutation k in clone c of cell s, and let $v_{sck}$ represent the corresponding variant allele frequency (VAF). Each mutation is associated with a latent variable $z_{\text{sck}}\in \{0,1\}$, where $z_{sck}=0$ corresponds to a mutation that contributes nothing to the disease score (zero-inflated component), while $z_{sck}=1$ denotes a mutation that contributes through the Gaussian component. The conditional distribution of $n_{sck}$ is therefore defined as

(1) $$P\left(n_{sck}|v_{sck},z_{sck},\pi_{sc},a_{sc},b_{sc},\sigma^{2}\right)=\left\{\begin{array}{c} \pi_{sc},\;\text{if}{\;z}_{sck}=0\\ \left(1-\pi_{sc}\right)\cdot N\left(n_{sck}|\mu_{sck},\sigma^{2}\right),\;\text{if}\;z_{sck}=1 \end{array}\right.$$

with mean structure

(2) $$\mu_{sck}=a_{sc}v_{sck}+b_{sc}$$

Here, $\pi_{sc}$ is the clone-specific zero-inflation probability, $a_{sc}$ denotes the selection pressure, $b_{sc}$ is the baseline effect, and $\sigma^{2}$ represents the residual variance of the Gaussian component. The joint likelihood over all cells, clones, and mutations is

(3) $$L\left(\pi_{sc},a_{sc},b_{sc},\sigma^{2},z_{sck}|n,v,z\right)=\prod_{s=1}^{s}\prod_{c=1}^{C_{s}}\prod_{k=1}^{K_{sc}}P\left(n_{sck}|v_{sck},z_{sck},\pi_{sc},a_{sc},b_{sc},\sigma^{2}\right)$$

where $n=\left\{n_{sck}\right\}$ denote the collection of disease relevance scores across all cells, clones, and mutations; let $v=\left\{v_{sck}\right\}$ denote the corresponding VAFs; and let $z=\left\{z_{sck}\right\}$ denote the latent indicators identifying whether each mutation contributes to the disease score.

To complete the model specification, we place hierarchical priors over all parameters. The zero-inflation probability $\pi_{sc}\sim \text{Beta}(\alpha ,\beta )$, the selection pressure $a_{sc}\sim \text{Beta}\left(\alpha_{a},\beta_{a}\right)$, and the baseline term $b_{sc}\sim N\left(0,\sigma_{b}^{2}\right)$. The residual variance follows an inverse-gamma distribution: $\sigma^{2}\sim Inv-\text{Gamma}(\kappa ,\theta )$. The posterior distribution is then proportional to the likelihood multiplied by the priors:

(4) $$P\left(\pi_{sc},a_{sc},b_{sc},\sigma^{2},z|n,v\right)\propto L\left(\pi_{sc},a_{sc},b_{sc},\sigma^{2}|n,v,z\right)P\left(z|\pi_{sc}\right)P\left(\pi_{sc}\right)P\left(a_{sc}\right)P\left(b_{sc}\right)P\left(\sigma^{2}\right)$$

Because this posterior does not admit a closed-form solution, inference is performed using a hybrid Markov Chain Monte Carlo (MCMC) algorithm that combines Gibbs sampling steps for parameters with conjugate updates and Metropolis–Hastings (MH) steps for those with non-conjugate full conditionals. The clone-specific zero-inflation probabilities $\pi_{sc}$ admit conjugate Beta updates. For each clone c in cell s, define

$$Z_{sc}=\sum_{k=1}^{K_{sc}}I\left(n_{sck}=0\right)$$

as the number of mutations with zero-valued disease scores, where $K_{sc}$ is the total number of mutations in that clone. Using the conjugacy between the Beta prior and Bernoulli likelihood, the posterior distribution of $\pi_{sc}$ is

$$\pi_{sc}|n_{sc}\sim \text{Beta}\left(\alpha +Z_{sc},\beta +K_{sc}-Z_{sc}\right)$$

In contrast, the clone-specific selection pressures $a_{sc}$ and baseline effects $b_{sc}$ do not yield conjugate posteriors and therefore require Metropolis–Hastings (MH) updates within the MCMC algorithm. For the selection pressure parameter $a_{sc}$, combining the Gaussian likelihood with the Beta prior leads to the following log-posterior:

$$\text{ln}\;\text{P}\left(a_{sc}|n_{sc},v_{sc},b_{sc},\sigma^{2}\right)\propto -\frac{1}{2\sigma^{2}}\left[a_{sc}^{2}\sum_{k=1}^{K_{sc}}v_{sck}^{2}-2a_{sc}\sum_{k=1}^{K_{sc}}v_{sck}\left(n_{sck}-b_{sc}\right)\right]+\left(\alpha_{a}-1\right)lna_{sc}+\left(\beta_{a}-1\right)\text{ln}\;\left(1-a_{sc}\right)$$

Because this density is non-standard, Gibbs sampling is not applicable. Instead, candidate values $a_{sc}^{\prime }$ are proposed, for example from a Beta distribution, and the acceptance probability is calculated as

$$R=\text{min}\left(1,\frac{P\left(a_{sc}^{\prime }|n_{sc},v_{sc},b_{sc},\sigma^{2}\right)}{P\left(a_{sc}|n_{sc},v_{sc},b_{sc},\sigma^{2}\right)}\right)$$

If the proposal is accepted, the parameter is updated by setting $a_{sc}=a_{sc}^{\prime }$; otherwise, the current value is retained. Repeating this procedure across MCMC iterations generates a Markov chain that converges to the posterior distribution of $a_{sc}$.

A similar strategy is required for the baseline parameter $b_{sc}$. The log-posterior for $b_{sc}$, derived from the Gaussian likelihood with a Gaussian prior, is given by

$$\text{ln}\;\text{P}\left(b_{sc}|n_{sc},v_{sc},a_{sc},\sigma^{2}\right)\propto -\frac{1}{2\sigma^{2}}\left[b_{sc}^{2}K_{sc}-2b_{sc}\sum_{k=1}^{K_{sc}}\left(n_{sck}-a_{sc}v_{sck}\right)\right]-\frac{1}{2\sigma_{b}^{2}}b_{sc}^{2}$$

Although this expression resembles the log-density of a Gaussian distribution, the presence of the prior term prevents closed-form conjugacy. Consequently, candidate values $b_{SC}^{\prime }$ are generated from a Gaussian proposal distribution, such as $N\left(b_{sc},\tau^{2}\right)$, and are accepted with probability

$$R=\text{min}\left(1,\frac{P\left(b_{sc}^{\prime }|n_{sc},v_{sc},a_{sc},\sigma^{2}\right)}{P\left(b_{sc}|n_{sc},v_{sc},a_{sc},\sigma^{2}\right)}\right)$$

Accepted proposals replace the current value of $b_{sc}$, while rejected proposals leave it unchanged. Through this combination of conjugate Gibbs updates for $\pi_{sc}$ and $\sigma^{2}$, and MH updates for $a_{sc}$ and $b_{sc}$, the MCMC algorithm generates samples from the joint posterior distribution in equation (4). This enables reliable inference of clone-specific selection pressures and baseline effects, as well as estimation of the global residual variance that captures unexplained heterogeneity in the MitoBayes model. For full derivations of the likelihood functions and posterior updates, please refer to the Supplementary Material.

### Simulation studies

To evaluate the robustness and parameter recovery capabilities of the proposed model, we generated synthetic data under controlled conditions that mirror biologically plausible patterns of cellular disease score formation. The simulation framework adheres strictly to the generative process defined in the model, capturing both clone-specific heterogeneity and stochastic zero-inflation commonly observed in mitochondrial mutation datasets. For each simulated cell sample, we assumed the presence of one clonal population, within which a fixed number of mitochondrial mutations were generated. Each mutation was assigned a VAF drawn independently from a uniform distribution over the unit interval, reflecting a broad range of allelic fractions observed in practice. Cellular disease score values were generated using a two-stage process. First, a latent binary variable was sampled to determine whether a mutation contributes to cellular disease score determination. Specifically, for each mutation, we sampled $z_{sck}\sim \text{Bernoulli}\left(1-\pi_{sc}\right)$, where $\pi_{sc}\in (0,1)$ represents the clone-specific probability of cellular disease score non-contribution due to biological factors or other mechanisms. When $z_{sck}=0$, the cellular disease score value was set to zero. Otherwise, when $z_{sck}=1$, the observed cellular disease score value was sampled from a Gaussian distribution with mean and standard deviation where captures the selection pressure exerted by the clone and denotes its baseline contribution to the cellular disease score. The term represents the Gaussian noise introduced to account for both biological variability and measurement uncertainty in cellular disease score assessment.

To validate the model, we simulated synthetic datasets as described in the Results (see Fig. 2). We generated cellular disease scores under known values and known π, adding noise, and then applied our inference to see if we recover the true parameters. We varied sample sizes (number of cell = 50, 100, … 500) and clone numbers, and also tested different prior settings to probe robustness. We computed mean absolute error (for point estimates) and credible interval coverage for and π. We also monitored MCMC diagnostics (trace plots, effective sample size, PSRF). All simulation tests demonstrated accurate recovery of parameters and well-behaved inference. The generated code for simulating data can be accessed in the public code repository.

### Differential expression and pathway analysis

In each case study, after obtaining clone-specific selection coefficients and per-cell selection pressure values, we stratified cells to compare those under high vs low selection pressure. For AD neurons, we took PVALB neurons and separated the top 50% vs bottom 50% of cells. We then performed differential gene expression analysis using the Wilcoxon rank-sum test (as implemented in Scanpy/Seurat) to identify genes significantly up- or down-regulated in high-pressure cells. We applied a false discovery rate (FDR) correction and considered genes with FDR < 0.05 and >1.5-fold change as significant. For NSCLC T/NK cells and HCC tumor cells, we did similar, comparing the high-selection subgroup vs the rest. Pathway enrichment analysis was conducted using clusterProfiler in R for KEGG pathways and Gene Ontology (GO) Biological Process terms. We supplied the list of significant DEGs (up or down) and used the built-in hypergeometric test with Benjamini–Hochberg correction for multiple pathways.

### Structural modeling of *MT-ATP6* mutation

To predict the impact of the *MT-ATP6* 8859A>G (Lys->Arg at position 52 of ATP6 protein) mutation, we performed in silico protein stability analysis. We retrieved the crystal structure of the human ATP synthase (or a high-quality homology model if crystal structure for subunit a was not available at needed resolution) and introduced the point mutation using PyMOL. We then used an energy calculation tool (FoldX) to estimate the change in folding free energy (ΔΔG) caused by the mutation. A positive ΔΔG indicates the mutant is less stable (higher energy) than wild-type, whereas a negative indicates a stabilizing mutation. The predicted ΔΔG of +1.14 kcal/mol for 8859A>G suggests a slight destabilization.

### Kaplan–Meier survival analysis

We leveraged The Cancer Genome Atlas (TCGA) to evaluate clinical correlations of mitochondrial gene expression. We obtained pan-cancer RNA-seq data and clinical outcomes for patients who had received chemotherapy (filtering for those with available survival information post-therapy). We focused on *MT-ATP6* expression as a proxy for mitochondrial OXPHOS activity. Patients were stratified into high vs low *MT-ATP6* expression groups (top quartile vs bottom quartile within each cancer type, to avoid tumor-type biases). We then performed Kaplan–Meier survival analysis using lifelines in Python, with overall survival (from start of therapy) as the endpoint. The log-rank test was used to assess differences between groups, and a Cox proportional hazards model was fitted to estimate the hazard ratio. The analysis (illustrated in Fig. 5h) showed a modest but significant survival disadvantage for patients with high *MT-ATP6*, consistent with our SCLC single-cell findings and previous reports that link OXPHOS-high states to treatment resistance.

## Supplementary Material

This is a list of supplementary files associated with this preprint. Click to download.

- SupplementaryMaterial.docx

## Data Availability

The single-cell transcriptome dataset of this study was obtained from the publicly available GEO database, including GSE157827 of AD (https://www.ncbi.nlm.nih.gov/geo/query/acc.cgi?acc=GSE157827), GSE164146 of NSCLC (https://www.ncbi.nlm.nih.gov/geo/query/acc.cgi?acc=GSE164146), GSE138474 of SCLC (https://www.ncbi.nlm.nih.gov/geo/query/acc.cgi?acc=GSE138474) and GSE112271 of HCC (https://www.ncbi.nlm.nih.gov/geo/query/acc.cgi?acc=GSE112271). The GWAS summary statistics used to calculate the disease-associated scores of cells are derived from the publicly available GWAS Catalogy database, and GCST007511 corresponds to late-onset Alzheimer’s disease (https://www.ebi.ac.uk/gwas/studies/GCST007511), GCST90479533 corresponds to lung cancer (https://www.ebi.ac.uk/gwas/studies/GCST90479533) and GCST90043858 corresponds to hepatocellular carcinoma (https://www.ebi.ac.uk/gwas/studies/GCST90043858).

## Code availability

All source codes and related tutorials have been made publicly available under the MIT License at Github (https://github.com/QSong-github/MitoBayes).
